# Vitamin D deficiency and associated factors in Jordan

**DOI:** 10.1177/2050312119876151

**Published:** 2019-09-13

**Authors:** Mohammed El-Khateeb, Yousef Khader, Anwer Batieha, Hashem Jaddou, Dana Hyassat, Nahla Khawaja, Mousa Abujbara, Kamel Ajlouni

**Affiliations:** 1 The National Center (Institute) for Diabetes, Endocrinology and Genetics (NCDEG), The University of Jordan, Amman, Jordan; 2 Jordan University for Science and Technology, Irbid, Jordan

**Keywords:** Vitamin D deficiency, prevalence, Jordan

## Abstract

**Background:**

In Jordan, many studies reported various rates of vitamin D deficiency and insufficiency among different groups. This study aimed to determine the prevalence of low vitamin D level among Jordanian adults and determine its association with selected variables.

**Methods:**

The vitamin D level was assessed in a national representative sample of 4056 subjects aged >17 years. The study involved face-to-face interviews with the subjects and measurement of serum 25(OH)D. Low vitamin D level was defined as 25(OH)D < 30 ng/mL. Deficiency was defined as 25(OH)D < 20 ng/mL, and insufficiency was defined as 25(OH)D level of 20–30 ng/mL.

**Results:**

The overall prevalence of low vitamin D status (25(OH)D < 30 ng/mL) was 89.7%, with higher prevalence in males (92.4%) than in females (88.6%). Vitamin D was sufficient in 7.6% of males, insufficient in 38.4% of males, and deficient in 54% of males. Among females, vitamin D was insufficient in 10.1% and deficient in 78.5%. The prevalence of vitamin D deficiency was much higher in females than in males (*p* = 0.001). The only variables that were significantly associated with low level of vitamin D were gender, age, obesity, and employment.

**Conclusion:**

The prevalence of low vitamin D level is extremely high in Jordan. Age, gender, obesity, and unemployment were associated with low levels of vitamin D. Health authorities in Jordan need to increase the level of awareness about vitamin D deficiency and its prevention, particularly among women.

## Introduction

Vitamin D deficiency is currently recognized as a common public health problem.^1^ The role of vitamin D in calcium and phosphate homeostasis and skeletal and non-skeletal health is well established. Vitamin D deficiency plays an important role among other factors in metabolic bone disorders leading to osteomalacia in adults and rickets in children.^2^ Vitamin D deficiency is associated with many chronic diseases including cardiovascular diseases, hypertension, diabetes, metabolic syndrome, depression, autoimmune diseases, cancer, neurocognitive function, and increased occurrence of infection.^3–7^

Almost 90% of the human endogenous vitamin D is synthesized primarily in the skin by activation of 7-dehydrocholesterol triggered by the exposure to ultraviolet (UVB) sunlight, so limited exposure to the sunlight might lead to vitamin D deficiency. The remaining 10% is acquired from nutritional sources such as codfish, mushrooms, milk, eggs, and fortified food. Although vitamin D synthesis depends on UVB sunlight exposure, many other factors such as age, obesity, skin color, dress style, and sunscreen-use might affect vitamin D level.^8^

In Arab countries, with sunlight throughout the year, vitamin D serum levels are expected to be adequate, yet studies from different Arab countries showed high prevalence of vitamin D deficiency and insufficiency.^9–14^ Globally, the prevalence of vitamin D deficiency is very high and varies from 70% to 90% in different populations.^15,16^

In Jordan, studies reported various rates of vitamin D deficiency and insufficiency among different groups. A 2009 national study showed that 37% of women were vitamin D deficient.^17^ Another study reported a much higher prevalence rate of vitamin D deficiency (62.3%) among women.^18^ This study aimed to determine the prevalence of low vitamin D level among Jordanian adults and determine its association with selected variables.

## Methods

### Sampling and data collection

This national cross-sectional study was conducted among Jordanian adults over a period of 4 months between May and August 2017. A population-based household sample was selected from 12 governorates covering the three regions of the country; the north, middle, and south. A multistage sampling technique was used to select the participants. A systematic sample of households from the catchment areas of 17 selected health centers was selected. A team of two members (a male and a female) visited the selected households and invited household members ⩾18 years of age to participate in the study and to report next morning to the health center after an overnight fast.

### Data collection and laboratory analysis

Trained persons interviewed the participants using a structured questionnaire. The questionnaire was developed by the research scholars to study various risk factors of cardiovascular diseases and nutritional status in Jordan including vitamin D deficiency. The same questionnaire was used in previous national surveys in 1994, 2004, and 2009. The questionnaire included sociodemographic variables and information on diabetes and other cardiovascular disease risk factors, morbidity, quality of life, and health services. Height, weight, waist and hip circumferences, and blood pressure were carried out in a standard way by trained research scholars. Three blood samples were drawn from a cannula inserted into the antecubital vein and used for different laboratory measurements. Samples were centrifuged within 1 h at the survey site, and transferred by separate labeled tubes in ice boxes to the central laboratory of the National Center of Diabetes, Endocrinology, and Genetics in Amman, Jordan. All biochemical measurements were carried out by the same team of laboratory technicians using the same method throughout the study period. 25-Hydroxyvitamin D (25(OH)D) was measured using the ARCHITECT 25-OH Vitamin D assay (Abbott Laboratories, Abbott Park, IL, USA). The assay is a delayed, one-step immunoassay with 6-point calibration. The method is used for the quantitative measurement of 25(OH)D in human serum and plasma samples. Low vitamin D level was defined as 25(OH)D < 30 ng/mL. Deficiency was defined as 25(OH)D < 20 ng/mL and insufficiency was defined as 25(OH)D level of 20–30 ng/mL.^19^ Body mass index (BMI) was calculated by dividing the weight (kg) by the height (m^2^). Participants with BMI of 30 kg/m^2^ or more were considered obese.

The study was approved by the Ethical Committee at the National Center for Diabetes, Endocrinology, and Genetics, Amman, Jordan. Written informed consent was obtained from each participant. All participants were informed that their information will be kept confidential.

### Statistical analysis

Data were described using mean values and percentages. Differences between proportions were tested using Chi-square test. Multivariable logistic regression analysis was performed to identify factors associated with low levels of vitamin D. For this purpose, vitamin D level was dichotomized, using 30 ng/mL as cutoff point, and entered into the model as a dependent variable. All other relevant variables were treated as independent variables. Variables showing no relationship to vitamin D status were excluded from the model, and the process was repeated until the best model was obtained. A *p*-value of less than 0.05 was considered statistically significant.

## Results

### Participant characteristics

This study included a total of 4056 subjects (1193 males and 2863 females), aged between 18 and 90 years. Table 1 shows the sociodemographic and relevant characteristics of the study participants. The mean age was 43.8 (SD = 14.3) years. The prevailing skin color of participants was wheatish (about 54.3%); 15.9% had dark skin, and the rest were whites or blondes.

**Table 1.** Distribution of the study population by sociodemographic characteristics.

Variable	No.	%
Age, years (mean = 43.7, SD = 14.2)		
18–39	1543	38.1
40–59	1922	47.5
60 and above	582	14.4
Gender		
Male	1191	29.4
Female	2856	70.6
Education, years of completed formal schooling		
Illiterate	210	5.3
1–11	1205	30.5
12	1112	28.2
>12	1424	36.0
BMI, kg/m^2^ (mean = 29.8, SD = 8.3)		
Underweight	93	2.3
Normal	810	20.2
Overweight	1293	32.2
Obese	1815	45.3
Marital status (*n* = 4038)		
Single	604	15.0
Married	3100	76.8
Divorced	64	1.6
Widowed	254	6.2
Separated	16	0.4
Occupational (*n* = 3952)		
Unemployed	2094	53.0
Retired	469	11.9
Indoor job	870	22.0
Outdoor job	519	13.1
Smoking status (*n* = 4027)		
Current smoker	584	14.5
Never smoked	3443	85.5
Skin color (*n* = 3908)		
Blonde and white	1164	29.8
Light brown	2121	54.3
Dark or black	623	15.9
Clothing style (*n* = 2764)		
Niqab	259	9.4
Scarf	2386	86.3
Western	119	4.3

SD: standard deviation; BMI: body mass index.

### Prevalence of low vitamin D

Table 2 shows the mean values of serum 25(OH)D according to gender and age. The mean values were significantly higher in males than in females across all age groups. The overall crude prevalence of low vitamin D status (25(OH)D < 30 ng/mL) was 89.7%, with higher prevalence in males (92.4%) than in females (88.6%). Vitamin D was sufficient in 7.6% of males, insufficient in 38.4% of males, and deficient in 54% of males. Among females, vitamin D was insufficient in 10.1% and deficient in 78.5%. The prevalence of vitamin D deficiency was much higher in females than in males (*p* = 0.001). The prevalence rates of low vitamin D level differed significantly according to age, years of education, marital status, and occupation among females only. (Table 3).

**Table 2.** Vitamin D status by age and gender (Jordan, 2016).

Age, years	*n*	25(OH)D (ng/mL), mean ± SD	25(OH)D level (ng/mL)			*p*-value
			<20	20–29.9	⩾30	
Gender						<0.001
Male	1183	20.2 ± 7.4	639 (54.0)	454 (38.4)	90 (7.6)	
Female	2771	15.1 ± 11.7	2176 (78.5)	279 (10.1)	316 (11.4)	
Total	3954	16.6 ± 10.8	2815 (71.2)	733 (18.5)	406 (10.3)	
Male						0.048
18–39	333	20.4 ± 6.4	170 (51.1)	140 (42.0)	23 (6.9)	
40–59	601	20.4 ± 7.4	317 (52.7)	239 (39.8)	45 (7.5)	
⩾60	249	19.2 ± 8.6	152 (61.0)	75 (30.1)	22 (8.8)	
Total	1183	20.2 ± 7.4	639 (54)	454 (38.4)	90 (7.6)	
Female						<0.001
18–39	1174	12.9 ± 10.1	1002 (85.3)	79 (6.7)	93 (7.9)	
40–59	1269	16.4 ± 12.3	944 (74.4)	153 (12.1)	172 (13.6)	
⩾60	328	17.8 ± 13.3	230 (70.1)	47 (14.3)	51 (15.5)	
Total	2771	15.1 ± 11.7	2176 (78.5)	279 (10.1)	316 (11.4)	

SD: standard deviation.

**Table 3.** Prevalence of low vitamin D (25(OH)D < 30 ng/mL) in the study population among males and females according to selected variables.

	Males			Females		
	*n*	%	*p*-value	*N*	%	*p*-value
Age, years			0.684			<0.001
18–39	309	93.1		1076	92.2	
40–59	555	92.5		1091	86.4	
⩾60	227	91.2		276	84.4	
Marital status			0.813			0.002
Ever married	958	92.3		2025	87.8	
Single	130	92.9		413	92.8	
Occupation			0.412			<0.001
Unemployed	132	93.6		1719	91.1	
Retired	297	90.3		99	74.4	
Office work	325	93.4		428	85.1	
Field work	325	92.6		138	86.8	
Body mass index			0.131			0.095
Normal	213	89.1		489	88.6	
Overweight	445	93.7		695	86.7	
Obese	414	93.0		1192	89.5	
Underweight	19	86.4		67	94.4	
Years of education			0.508			0.001
Illiterate	12	100.0		169	92.3	
1–11	321	90.9		742	89.3	
12	325	92.1		666	91.2	
>12	406	93.1		816	85.7	
Smoking			0.346			0.346
Yes	366	93.4		167	91.3	
No	718	91.8		2266	88.4	

### Multivariate analysis of factors associated with low vitamin D

The multivariate analysis of factors associated with low level of vitamin D is shown in Table 4. The only variables that were significantly associated with low level of vitamin D were gender, age, obesity, and employment. The odds of low vitamin D level among women was almost twice that odds for men (odds ratio (OR) = 2.11). People aged 40–59 years had higher odds of low vitamin D level (OR = 2.56) compared to those who were younger than 40 years. Obesity was associated with increased odds of low vitamin D level (OR = 1.52). Unemployed people had higher odds of low vitamin D level (OR = 1.55) compared to employed people.

**Table 4.** Factors independently related to low vitamin D, using multivariate logistic regression analysis.

Variable	OR (95% CI)	*p*-value
Gender		
Male	1	
Female	2.11	<0.001
Age, years		
18–39	1	
40–59	2.56	<0.001
60 and above	1.31	0.085
Body mass index, kg/m^2^		
Normal	1	
Overweight	1.20	0.224
Obese	1.52	0.005
Occupation		
Employed	1	
Unemployed	1.55	<0.001

## Discussion

The prevalence of vitamin D deficiency is alarming high globally.^20^ Previous studies in Jordan revealed various rates of vitamin D deficiency.^17,18,21^ In this study, 89.7% of Jordanian adults had low vitamin D level (25(OH)D < 30 ng/mL). Nichols et al.^21^ reported that 60.3% of non-pregnant women of childbearing age were vitamin D deficient. This estimate was much higher than what had been reported by Batieha et al.^17^ in a previous national study who reported a prevalence of 37.3% in females and 5.1% in males.

It is well-known that many methods have been developed by several companies to measure 25(OH)D levels in serum or plasma. Numerous publications have shown the limitations of these methods, with notable variations in vitamin D determination by using these different assays.^22,23^ The differences in the measurements of 25(OH)D levels because of using different assays might explain the variations in the prevalence rates. To investigate the differences in the findings of this study and the findings of a previous national study by Batieha et al.,^17^ we re-measured the level of vitamin D in 1000 stored samples from Batieha et al.^17^ study using Abbott reagents (Abbott Laboratories) instead of the previously used radioimmunoassay (RIA). We found that 91% of the study group had low vitamin D level.

Moreover, high prevalence rates of low vitamin D were reported in other countries of the region including Lebanon, 44%;^8^ Syria, 90.1%;^9^ Saudi Arabia, 83.6%;^10^ Qatar, 90.4%;^11^ Morocco, 91%;^12^ Tunisia, 47.6%;^13^ and Egypt, 60%.^14^

The main factors that were significantly associated with low level of vitamin D in the multivariate analysis in our study were gender, age, obesity, and employment. Vitamin D deficiency and insufficiency affected all age groups in this study. Females were twice more likely than men to have low level of vitamin D. Although Jordan is a sunny country almost all of the year, the high rate among females might be explained by that they are not receiving enough exposure to sun because of their dressing style. In this study, 95% of women were either veiled, wearing the *niqab* (which covers the whole body including the face and hands), or wearing the *hijab* (which covers the whole body but spares the face and hands). The finding of a higher prevalence of low vitamin D status in women wearing the niqab or hijab compared to women wearing Western dress styles is consistent with previous studies.^17,18^ The prevalence of hypovitaminosis reported by Mishal^18^ study was 31% in Western-dressed women, 55% in women wearing the hijab, and 83% in women wearing the niqab.

The findings of our study supported the association between obesity and low vitamin D levels. Previous studies showed that obesity was associated with lower serum 25(OH)D concentrations.^24,25^ However, the mechanism explaining this association is not fully described. One possible explanation is that people with obesity are less likely to participate in outdoor activities and more likely to cover-up and wear more clothing than leaner individuals, thus decreasing sun exposure and limiting endogenous production of cholecalciferol in the skin.

The link between vitamin D levels and occupation has previously been explored. Low levels of vitamin D have been reported in occupational groups with low exposure to sunlight^26^ and high levels were reported among outdoor workers.^27^ In our study, unemployment was significantly associated with low vitamin D level. Unemployed people in Jordan, especially women, spend less time outdoors and therefore they are less likely to be exposed to the sun.

The main limitation of this study is inherited in its cross-sectional design. Temporal association and causality cannot be established in cross-sectional studies. Measurement errors in dealing with anthropometric measurements and vitamin D level might affect the study findings. A prospective study is strongly recommended to determine the true risk factor of low vitamin D level.

In conclusion, the prevalence of low vitamin D level is extremely high in Jordan. Age, gender, obesity, and unemployment were associated with low levels of vitamin D. Health authorities in Jordan need to increase the level of awareness about vitamin D deficiency and its prevention, particularly among women.
